# Effect of elevated levels of CO_2_ on powdery mildew development in five cucurbit species

**DOI:** 10.1038/s41598-020-61790-w

**Published:** 2020-03-19

**Authors:** Mujeebur Rahman Khan, Tanveer Fatima Rizvi

**Affiliations:** Department of Plant Protection, Faculty of Agricultural Sciences, Aligarh Muslim University, Aligarh, 202002 India

**Keywords:** Climate change, Environmental impact

## Abstract

The environment is the key factor that influences the host-parasite relationship. Elevated CO_2_ levels resulting from various anthropogenic sources may directly affect the surroundings around pathogens and plants. It is hypothesized that plants may respond differently to pathogens in the environment containing an elevated concentration of CO_2_. To test the hypothesis an experiment was conducted to examine the effects of intermittent exposures of elevated levels of CO_2_ *viz*., 400, 500 and 600 ppm (5 hr/day on alternate days) on the development of *Sphaerotheca fuliginea* causing powdery mildew disease on five cucurbits species using open-top chambers. The elevated levels of CO_2_ acted as a growth promoter and significantly enhanced the plant growth of all five cucurbit species. Inoculation with the fungus incited specific mildew symptoms on the leaves and decreased the plant growth and biomass production of the cucurbits tested except bitter gourd. The intermittent exposures with elevated levels of CO_2_ aggravated the disease development. As a result, severe mildew developed on all five cucurbits, including bitter gourd, which expressed tolerance to the pathogen. Fungus colonization in terms of the number of conidia/cm^2^ leaf surface was significantly greater on the plants exposed to 500 or 600 ppm CO_2_. The stomata and trichome density and stomatal pore width were increased in the leaves of CO_2_ exposed plants. The CO_2_ exposures also accelerated the photosynthesis rate, but transpiration, stomatal conductance, salicylic acid and total phenols were decreased; fungus inoculation caused the effects just reverse of CO_2_. Interaction between *S. fuliginea* and CO_2_ was found synergistic at 500 ppm, whereas with rest of the concentrations it was near to additive.

## Introduction

Air is an important vital resource for the sustenance as well as the development of living organisms. The composition of minor constituents of air may vary as a result of the emissions emerging from various industrial and urban activities^1^. In developing countries, fossil fuel continues to serve ae the main source of energy to run industries which causes emission of high concentration of CO_2_. This has largely contributed to a rise of around 28% CO_2_ of the existing 400+ ppm of the global mean ambient CO_2_ concentration in comparison to 311 ppm used to prevail during the middle of the nineteenth century (NASA GISS)^2^. If the existing trend of fossil fuel burning and human population rise continues, the ambient CO_2_ levels may have reached 500–1000 ppm by the year 2100^3^.

The elevated concentrations of CO_2_ may influence plant growth and host-pathogen relationship^4^. Carbon dioxide is not a phytotoxic gas; rather it may promote the plant growth and biomass production^5^. The plant growth promotion occurs due to enhancement in CO_2_ fixation rate through a check on photorespiration^6^. The accelerated rate of photosynthesis under elevated CO_2_ levels generally leads to enhanced plant growth and biomass production. The plant growth-promoting effect of CO_2_ varies with the physiological group of the plant *i.e*., 30–34% in C_3_ plants (the majority of plants) and 10–15% in C_4_ plants (members from Poaceae)^7,8^. Besides, CO_2_ may also influence other physiological and biochemical functions of the exposed plants^9,10^. The stomatal conductance and transpiration are negatively influenced by higher concentrations of CO_2_^11^. Likewise, elevated levels of CO_2_ (500–600 ppm) enhance the synthesis of chlorophylls and other leaf pigments^12^.

Plants are generally attacked by several pests and pathogens, and exhibit a substantial decrease in the plant growth and biomass production, depending on the disease severity^13^. Elevated levels of CO_2_ and related climatic change can influence the virulence of plant pathogens and host-parasite relationship^14^. These CO_2_-mediated effects may promote the disease development^15^ and aggravate the disease severity^14^. Plant-pathogen interactions under elevated levels of CO_2_ have the potential to interrupt both natural and agricultural pathosystems. Lake and Wade^15^ reported that the powdery mildew fungus, *Erysiphe cichoracearum* on *Arabidopsis thaliana* became more aggressive at 800 ppm of CO_2_, and also caused variations in the leaf epidermal traits. The length of the guard cell, number of trichomes, and stomatal density were found to be increased in the leaves of plants exposed to elevated CO_2_ levels. Pugliese *et al*.^16^ also evaluated the effects of elevated CO_2_ levels on the powdery mildew fungus *Podosphaera xanthii* infecting zucchini (*Cucurbita pepo*), planted in four different simulated climatic conditions *viz*., 450 and 800 ppm of CO_2,_ at elevated temperatures (28 °C day, 22 °C night). The combination of elevated CO_2_ and temperature invariably favored the powdery mildew development and enhanced the disease severity on zucchini as compared to control.

Although limited investigations have been carried out to study the plant disease development under elevated CO_2_ levels, the information is sufficient to indicate that the higher concentrations of CO_2_ may influence the development of plant diseases caused by fungi. The present investigation was done to ascertain the effect of elevated levels of CO_2_ relevant to existing and future projected ambient concentrations on the development of powdery mildew caused by *Sphaerotheca fuliginea* on five common cucurbits, namely pumpkin, bottle gourd, sponge gourd, cucumber and bitter gourd were examined in open-top chambers. Besides morphological variables, important physiological (photosynthesis rate and transpiration, stomatal conductance), biochemical (salicylic acid, total phenol, chlorophyll a, and carotenoids) and anatomical parameters (stomata and trichomes) were also studied in relation to CO_2_ exposure and fungus inoculation.

## Materials and Methods

### Exposure system

**Figure 1:**
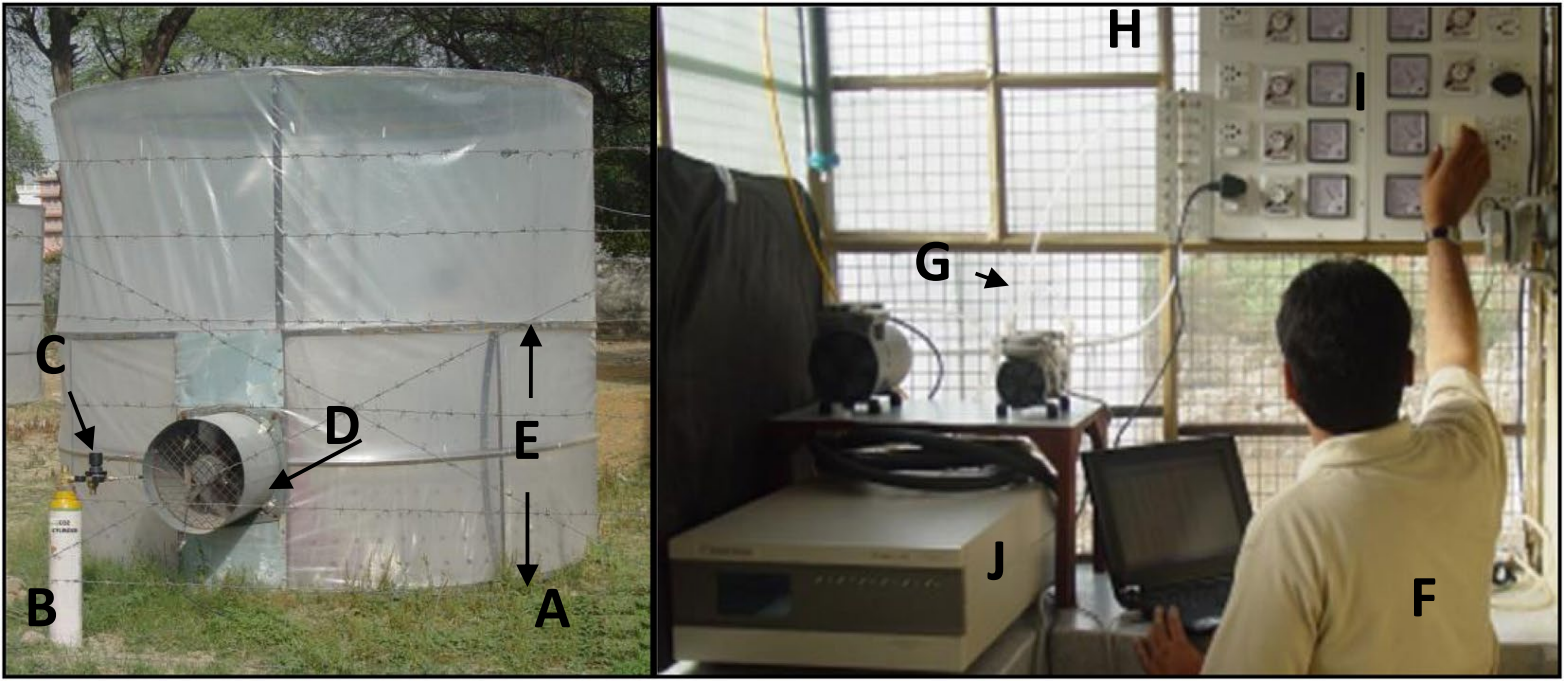
(**A**) Open-top exposure chambers used to expose plants to CO_2_; (**B**) CO_2_ Gas cylinder; (**C**) Double stage gas regulation; (**D**) Blowing assembly; (**E**) Double layer wall with perforations on the inner wall; (**F**) Gas monitoring and regulating assembly; (**G**) Teflon pumps to supply CO_2_-air mixture to the Analyzer; (**H**) Anemometer; (**I**) Panel to regulating voltage supply to the blowers; (**J**) CO_2_ Gas analyser.

Plants were exposed to CO_2_ using open-top cylindrical exposure chambers of 2 × 2.5 m dimension (diameter × height; Fig. 1). These chambers were dynamic in state, without having a stagnation of air in any part of the chamber. The average rate of air flow inside the chamber at different heights was 0.2–0.5 m/s, and the total replacement of the air inside the chamber took place once in 1.0–1.5 min. The chambers were made of transparent poly-sheets with open-top. Therefore, any substantial interference in the sunlight incidence was not recorded. The wall in the lower half of the chambers was two-layered, and the inner wall was perforated to introduce CO_2_ air mixture into the chamber. The CO_2_ gas was introduced into the exposure chambers through the blowing assembly from a gas cylinder filled with 95–98% CO_2_ + 2–5% N_2_ (Sigma Gases, New Delhi; Fig. 1). The concentration of CO_2_ inside the chamber during exposure of plants was observed through the CO_2_ analyzer (EC9820 Ecotech, Australia; Fig. 1). Just a day before the start of exposures. The analyzer was calibrated to build the relationship between analyzer’s response and CO_2_ levels by the multipoint calibration system over the full range of the analyzer as per the operation manual of Ecotech.

Three inlet Teflon pipes were fitted to measure the CO_2_ level inside the chamber at 1, 2 and 3 ft. heights, which supplied the air-gas mixture with the help of a Teflon suction pump to the CO_2_ analyzer. At these heights, 1–3% variation in the CO_2_ concentration was recorded. During the exposure of plants, sampling of CO_2_ inside the chamber was done from the above three heights to minimize locational variation in the CO_2_ level. The frequency of CO_2_ monitoring in every chamber was once in 45 min for 5 min/sampling. An exhaust pump was fitted to the exhaust orifice of CO_2_ analyzer to drain out of the air-gas mixture. The desired level of CO_2,_ *i.e*., 400, 500 and 600 ppm (5 h mean) was maintained by regulating the flow rate of CO_2_ from the cylinder and the speed of the blowing assembly. The concentration monitoring and regulating system has been described elsewhere^17^. The ambient concentrations of SO_2_, O_3_ and SPM at the experimental site were also monitored (7 h once in a week)^17,18^. These gases and SPM were present within permissible limits for agriculture areas (<5 ppb and 50 µg/m^3^) at the experimental site during the experiment, and the probability of their interference with CO_2_ was negligible.

In total, eight exposure chambers of the same dimension were used. The chambers were placed in an open field with a spacing of 7–8 meters to avoid re-entering of CO_2_-air mixture ventilating from the open top. The location of the experiment was 27°55′23.6″N 78°04′29.2″E and the climate was semi-arid type in general. Eight treatments, *i.e*., four CO_2_ concentrations with powdery mildew fungus inoculation and the same four CO_2_ concentrations without the fungus inoculation were maintained. The 8 treatments include the uninoculated control plants (without *S. fuliginea*) and inoculated control plants (with *S. fuliginea*) which were exposed to ambient air (containing ~375 ppm CO_2_) inside two similar exposure chambers for similar air flow rate and duration. After exposure, the pots were left inside the chambers. However, before start of each exposure, the pots were rotated among the chambers to exclude the effect of chamber variation as only 8 chambers were available for 8 treatments. This method is a good substitute of chamber replication^17,19^.

### Inoculation with *Sphaerotheca fuliginea*

Leaves of the five test crops were inoculated with the source leaves (pumpkin) infected with *S. fuliginea* by the modified leaf rolling method^20^. The pure culture of *S. fuliginea* was maintained on pumpkin in a poly house. The pumpkin leaves having at least 25% leaf surface colonization with *S. fuliginea* were rolled over the leaves of the test plant at three weeks old stage. The inoculation was done daily continuously for three days.

### Collection of germplasm and plant culture

The indigenous germplasm of five cucurbits *viz*., pumpkin (*Cucurbita pepo* L.), bottle gourd (*Lagenaria siceraria* (Mol.) Standl.), sponge gourd (*Luffa cylendrica* (L) M. Roemd.), cucumber (*Cucumis sativus* L.) and bitter gourd (*Momordica charantia* L.) were purchased from an authorized seed dealer, M/S Chola Beej Bhandar, Aligarh, India. The response of these cucurbits to CO_2_ exposure or *S. fuliginea* infection was not known. All the seeds were surface sterilized with 0.5% NaOCl. Seeds were sown in the autoclaved mixture of field soil and compost (3:1 ratio) in 15 cm diameter clay pots (4 seeds/pot). Seedlings were thinned after four weeks of sowing in order to maintain one seedling/pot. The pots were then transferred to the exposure chambers and inoculated with the powdery mildew fungus. Five replicates were maintained for each treatment of a cucurbit species. Pots were watered with 250 ml tap water on alternate days. The intermittent exposures on alternate days started two days after the third inoculation of plants with *S. fuliginea* and lasted for three months with a total of 46 exposures of 5 h duration each. The plants were observed regularly throughout the study for the appearance of mildew symptoms, CO_2_ effects, etc. Plants were harvested after four months of sowing, and the gas injury, if any and powdery mildew disease severity, leaf colonization, plant length, rate of photosynthesis and transpiration, stomatal conductance, salicylic acid contents, total phenols, leaf pigments (chlorophyll a and carotenoids) and colonization of *S. fuliginea* were determined.

### Disease severity and leaf colonization of *S. fuliginea*

The powdery mildew disease severity was evaluated in terms of percent leaves of the plant showing mildew symptom. The percent colonization of *S. fuliginea* on cucurbit leaves was determined by percent leaf area covered by the powdery mass (conidia and mycelium). To measure the leaf area, a transparent graph paper was placed on the leaf and squares (0.25 cm^2^) covering the entire leaf as well as the powdery areas were counted. The partial squares were counted if at least half was covered by the mildew or leaf. The squares covered with less than half colonized area were not counted.

### Estimation of chlorophyll and carotenoids

The chlorophyll a and carotenoids content of leaves of the cucurbit species were determined by crushing 1 g of fresh leaves from interveinal areas in 40 ml of 80% acetone with the help of a mortar and pestle. The suspension was then transferred in a Buchner funnel having Whatman filter paper no.1. The filtration was done with the help of a suction pump. The residue was crushed three times by adding acetone. The suspension was transferred in the Buchner funnel. Therefore, the mortar and pestle were rinsed with acetone and the solution was transferred to the funnel. The active solution was filtered in vacuum. The filtrate was decanted in a 100 ml volumetric flask. The volume was made up to the capacity by adding acetone. The optical density (O.D) of the filtrate was read in a UV-spectrophotometer (Shimadzu, Japan) at 480 and 510 nm for carotenoids, and 645 and 663 nm for the chlorophyll^21^.

### Conidial size, germination and fibrosin bodies

The conidia of *S. fuliginea* were dusted on clean glass slides from the infected leaves of cucurbit plants to measure their size (length and width) under a microscope. To determine the conidial germination, some extra slides dusted with the conidia were placed on glass triangles kept in Petri plates filled with sterilized water. The plates were covered with a lid lined with moisturized cotton and incubated at 25 ± 2 °C for 36 hrs. After incubation, microscopic examination of the slides was done. The fibrosin bodies were counted from the freshly dusted conidia mounted in 3% aqueous potassium hydroxide solution. The observations on above conidial characters were taken from 100 conidia/treatment.

### Determination of number and size of stomata and number of trichomes

Fresh and fully-grown leaves of relatively same age and size were fixed in formalin aceto-alcohol (FAA) and preserved in 70% ethyl alcohol. The preserved leaves were cut into 1-cm^2^ size pieces which were boiled in 4% HNO_3_ to remove the epidermal peels (Ghouse & Yunus, 1972). The peels were then washed in tap water and stained in iron-alum and hematoxylin. Thereafter, the peels were dehydrated in an ethyl alcohol series and mounted in Canada balsam for examination under a microscope. The number of trichomes and stomata per l-cm^2^ peel were counted, and the size of stomatal aperture was measured.

### Estimation of foliar salicylic acid and phenolic compounds

Leaf samples from each treatment of a cucurbit species were collected and cut into 0.5–1.0 cm pieces. 1 g pieces were soaked overnight in water. The suspension was filtered through the Whatman filter paper no.1. The ethyl acetate fraction was taken and Na_2_SO_4_ was added to eliminate the water content and the filtrate was evaporated in a water bath. Methanol (10 ml) was added to the dried sample and solution was kept for observing the absorbance at 306 nm through a spectrophotometer^16^ (UV 2450, Shimadzu Japan). Standard curve of salicylic acid was prepared by salicylic acid concentrations *viz*., 0, 10, 20, 30, 40, 50 and 100 ppm in methanol. From the standard curve the concentration of salicylic acid in the sample was calculated with the help of the formula, y = mx ± c^22^, where, x and y denotes axis, m denotes gradient and c denotes y-intercept.

The total phenolic content of the leaf extract was evaluated through a modified Folin-Ciocalteu method^23^. The extract was diluted in distilled water (0.4 mg/5 ml). The diluted solution (0.5 ml), was mixed with Folin-Ciocalteu reagent (2.5 ml, 1/10). The mixture was left to settle for 2 mins at room temperature and 2 ml of aqueous sodium carbonate solution (75 g/L) was then added to the solutions. The combination was mixed thoroughly and incubated at 50 °C for 15 min and then cooled in an ice-water bath. Distilled water alone was used as a blank. The absorbances were measured in a UV spectrophotometer (Shimadzu Japan) at 760 nm. Gallic acid (1–8 µg/ml) was used to prepare a calibration curve. The phenolic contents were expressed in mg of Gallic acid equivalents/g of dry extract according to the calibration curve, y = 0.133x + 0.001 with correlation coefficient (r^2^) of 0.999, where y denotes absorbance and x denotes gallic acid concentration in mg/l.

### Physiological parameters

Portable Photosynthesis System (LI6400XT, Li-Cor, USA) and PAM Fluorescence Analyzer (PAM, Walz, Germany) were used to measure the rate of photosynthesis, transpiration and stomatal conductance. These parameters are important to determine overall impacts of CO_2_ on plant physiology. The above parameters were determined 1 hr before, during (2–3 hrs after start of an exposure) and 1 hr after completion of each intermittent CO_2_ exposures weekly throughout the experiment.

### Statistical analysis

The experiment with similar treatments was repeated over two successive years to assess the reproducibility of results. The effects of fungus inoculations and CO_2_ concentrations on the variables considered in the study were found statistically identical over two consecutive years at P ≤ 0.05. Therefore, the data of the two years were pooled. The means of 10 replicates (5 replicates/year) taken for each treatment were calculated and presented in tables and figures. The data (10 replicates) were subjected to a two-factor analysis of variance. The CO_2_ concentrations (4 treatments *i.e*., 375, 400, 500 and 600 ppm) was considered as factor-one and inoculation with the powdery mildew fungus as factor-two (2 treatments). Contrasts corresponding to CO_2_ levels and their interactions with fungus, pair-wise comparison Tukey honest significance difference method was used. The complete analysis was done through the “R” software package^24^. The data on plant length, rate of photosynthesis, rate of transpiration, stomatal conductance, salicylic acid content, phenolic content, chlorophyll content, carotenoids, number of trichomes and stomata, size of stomatal aperture and trichome length were subjected to a three-factor analysis and LSD was calculated to identify significant treatments at P ≤ 0.05 probability level using the package Agricolae of R software^25^. The effect of CO_2_ levels on disease severity, fungal colonization, conidial size (length and width) and number of fibrosin bodies were calculated by two factors ANOVA to observe significant treatments at the probability level, P ≤ 0.05. The figures showing contrast or differences in mean levels of interaction and individual effects of the two factors were generated through the R software. Percent decrease or increase as compared to control was evaluated and used to describe the results.

## Results

### Symptoms

**Figure 2:**
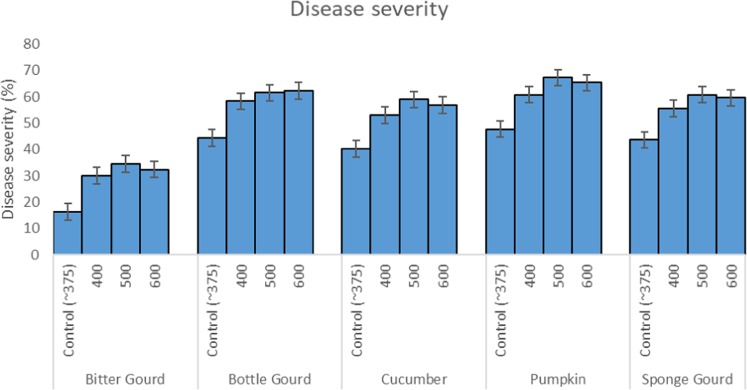
Bar diagram showing % disease severity in five cucurbit species exposed to elevated concentrations of CO_2_.

The symptom of powdery mildew disease in the form of circular, small, white, powdery mass (conidia and mycelia) developed first on the older leaves of all five cucurbit species. The white powdery colonies were occasionally seen on the stems. The number and size of the colonies later increased and eventually coalesced covering a large part of the leaf surface. Severely infected leaves slowly turned yellowish and withered. Such leaves later died and dried. The order of disease severity in cucurbit species tested was pumpkin (47%) > bottle gourd (44%) > sponge gourd (43%) > cucumber (40%) > bitter gourd (16%) (Fig. 2).

**Figure 3:**
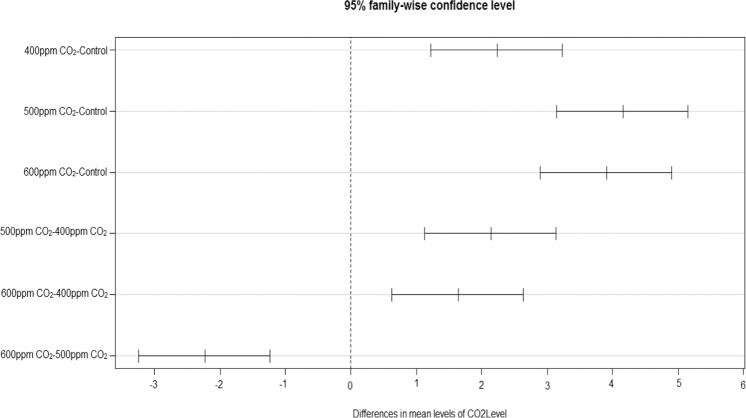
Effects of intermittent exposures of to 375 (ambient), 400, 500 and 600 ppm CO_2_ on the disease severity of the powdery mildew fungus, *S. fuliginea* on cucurbits. The contrast located on the left side of the line of no difference shows negative interaction (antagonistic), whereas the contrasts on the right side of the line of no difference represent positive interaction (synergistic). The contrast placed at the farthest location shows maximum effect of interaction.

The CO_2_ exposures did neither cause any toxicity symptom nor affect the time of appearance of the mildew symptoms. However, the disease severity was considerably higher in CO_2_ exposed plants (Fig. 2). The plants intermittently exposed to CO_2_ at 500 and 600 ppm showed significantly greater mildew severity and colonization of *S. fuliginea* on the leaves of all five cucurbit species in comparison to ambient or 400 ppm concentration. Maximum enhancement in the disease severity and fungal colonization was observed at 500 ppm CO_2_ concentration followed by 600 and 400 ppm in comparison to ambient air. Disease severity in 500 ppm CO_2_ exposed plants was recorded greatest on pumpkin (67%), followed by bottle gourd (61%), sponge gourd (60%), cucumber (58%) and bitter gourd (34%) due to exposure to 600 ppm CO_2_ (Figs. 2 and 3). The Fig. 3 on the orthogonal contrasts between CO_2_ levels shows that the interaction between CO_2_ and powdery mildew fungus was mostly synergistic and all contrasts except the 600 ppm:500 ppm CO_2_ contrasts. The contrasts except for 600 ppm:500 ppm were located in the zone of positive interaction on the right side of the line of no difference (Fig. 3). The figure also expressed the highest stimulatory effect of 500 ppm CO_2_ on the disease severity, as the contrast for this concentration (500 ppm:0 ppm) was placed on the farthest location from the line of no difference (Fig. 3). The contrast 600 ppm:500 ppm CO_2_ was observed in the region of negative interaction (antagonistic) *i.e*., on the left of the line of no difference (Fig. 3).

### Plant growth

**Figure 4:**
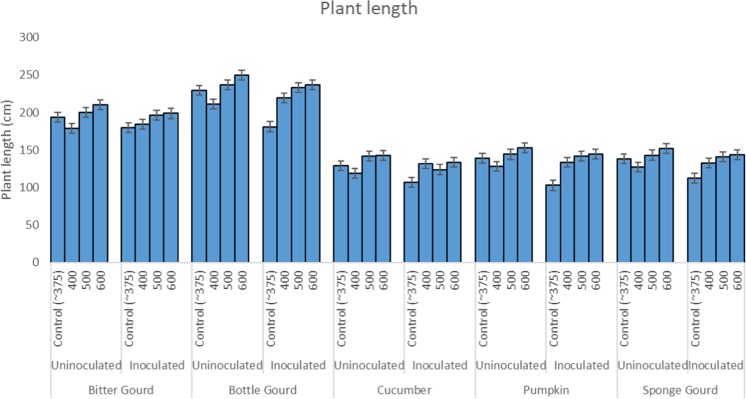
Bar diagram shows the effect of CO_2_ exposures and *S. fuliginea* inoculation singly and concomitantly on the plant length of five cucurbits.

The inoculation with *S. fuliginea* significantly decreased the length and dry weight of all five cucurbit species except bitter gourd as compared to uninoculated control (P ≤ 0.05). The suppression in the plant growth was in the order of pumpkin > bottle gourd > sponge gourd > cucumber > bitter gourd as compared to control (Fig. 4). The ANOVA revealed significant individual effects of the fungus on the above two variables at P ≤ 0. 001. Intermittent exposures of CO_2_ enhanced the plant growth and biomass of plants (Fig. 4). The enhancement in the plant growth variables was recorded significant for all five cucurbit species tested at 500 and 600 ppm CO_2_ in comparison to 400 ppm CO_2_ or control (ambient air ~375 ppm, Fig. 4). The increase in the plant growth at 600 ppm CO_2_ was relatively greater than 500 ppm concentration. The individual effects of CO_2_ at P ≤ 0.05 on the plant growth and powdery mildew fungus were significant at P ≤ 0.001.

The inoculated plants that were exposed to CO_2_ exhibited enhancement in the plant growth (shoot length) that varied with the CO_2_ concentration (Fig. 4). The interaction between *S. fuliginea* and 600 ppm CO_2_ was observed near to additive, and that led to a suppression in plant length close to the sum of increase or decrease caused by 600 ppm CO_2_ and fungus individually (Fig. 4). For example, 600 ppm CO_2_ enhanced the plant length by 16%, whereas *S. fuliginea* inoculation reduced by plant length by 12% and the sum of these effects comes 4%. The combined treatment of 600 ppm CO_2_ + *S. fuliginea* resulted in an increase of 3% in the plant length. An almost similar interaction was also recorded for 400 ppm CO_2_ and the fungus. However, at 500 ppm CO_2_, the interaction with *S. fuliginea* was synergistic e.g., 500 ppm CO_2_ increased the plant length by 12% while the mildew disease caused a decrease of 12%, hence net effects come 0.0%. However, in the combined treatment, 11% decrease in plant length occurred which is significantly greater than the additive effect at P ≤ 0.05.

**Figure 5:**
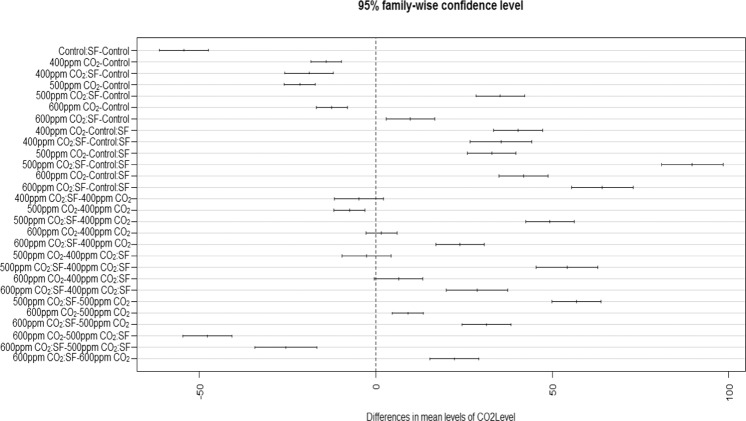
Effect of intermittent exposures of CO_2_ at 375 (ambient), 400, 500 and 600 ppm and *S. fuliginea* on the plant length of cucurbits. The contrasts situated on the left side on the line of no difference show negative interaction (antagonistic), whereas the contrasts located on the right side of the line of no difference represent positive interaction (synergistic). The contrasts situated at the farthest location shows highest interaction effect.

Figure 5 prepared on orthogonal contrasts between CO_2_ levels and fungus has shown a mostly significant interaction between CO_2_ and *S. fuliginea* as only a few contrasts lied on the broken vertical line of no difference. Maximum synergistic interaction with regard to suppression in the plant growth was observed for 500 ppm CO_2_ *S. fuliginea*: 0 ppm CO_2_ *S.* *fuliginea*, as this contrast was situated at the farthest location from the line of no difference (Fig. 5). The interactive and individual effects of CO_2_, crops and *S. fuliginea* were significant at P ≤ 0.001 (Tables 1–5).

**Table 1:** Effect of elevated levels of CO_2_ on colonization, conidia and fibrosin bodies of *S. fuliginea*, cuticular traits, physiological and biochemical parameters of bitter gourd inoculated with the powdery mildew fungus.

CO_2_ Concentrations (ppm)	*S. fuliginea*.	Colonization (%)	Trans piration (mmol H_2_O m^−2^ s^−1^)	Stomatal Conductance (mol m-2 s^−1^)	Phenolic contents (µg g^−1^ fresh leaf)	Carotenoids (µg g^−1^ fresh leaf)	Conidial size (µm)		Fibrosin bodies/conidia	Trichomes /cm^2^	Stomata/cm^2^	Size of stomatal aperture (µm)	Trichome length (µm)
							length	width					
Control (ambient)	Un inoculated		2.101	1.919	121.95	39.42				121.33	95.36	5.75	98.84
400			2.027	1.852	120.41	39.89				123.03	97.06	5.01	101.6
500			1.908	1.742	120.15	42.46				124.33	97.94	4.97	112.34
600			1.838	1.679	118.86	43.20				124.87	99.02	4.31	118.67
Control	Inoculated	47.74	2.273	2.076	132.65	36.27	27.31	11.31	5.63	117.54	90.133	6.24	90.07
400		49.41	2.200	2.009	135.00	36.35	28.18	11.67	5.81	118.83	91.36	5.97	94.23
500		53.80	2.078	1.898	137.45	38.91	28.27	11.71	5.83	119.04	92.05	5.48	98.98
600		52.75	2.011	1.836	139.67	39.74	28.35	11.74	5.84	119.93	92.96	5.02	102.04
**LSD (P** ≤ **0.05)**													
**Treatment**		—	0.04	0.04	2.55	1.36	—	—	—	2.53	2.24	0.11	2.12
**CO**_**2**_**Level**		2.22	0.06	0.06	3.61	1.93	1.01	0.71	0.68	3.59	3.16	0.15	3
**CO**_**2**_**Level:Treatment**		—	0.09	0.08	5.1	2.72	—	—	—	5.07	4.48	0.22	4.25

**Table 2:** Effect of elevated levels of CO_2_ on colonization, conidia and fibrosin bodies of *S. fuliginea*, cuticular traits, physiological and biochemical parameters of bottle gourd inoculated with the powdery mildew fungus.

CO_2_ Concentrations (ppm)	*S. fuliginea*.	Colonization (%)	Trans piration (mmol H_2_O m-2 s^−1^)	Stomatal Conductance (mol m-2 s^−1^)	Phenolic contents (µg g^−1^ fresh leaf)	Carotenoids (µg g^−1^ fresh leaf)	Conidial size (µm)		Fibrosin bodies/conidia	Trichomes /cm^2^	Stomata/cm^2^	Size of stomatal aperture (µm)	Trichome length (µm)
							length	width					
Control (ambient)	Un inoculated		1.913	1.712	109.21	31.13				107.31	80.22	2.64	79.23
400			1.854	1.659	108.11	31.47				111.07	83.03	2.12	81.07
500			1.739	1.556	107.32	33.50				115.79	86.56	1.98	88.26
600			1.678	1.501	106.02	34.09				117.50	87.84	1.27	93.54
Control	Inoculated	14.52	2.072	1.854	125.23	28.39	26.31	10.54	5.21	99.91	74.68	2.96	71.52
400		29.94	2.012	1.801	128.57	28.73	27.15	10.88	5.38	103.77	77.57	2.43	76.46
500		32.59	1.898	1.698	129.88	30.76	27.23	10.91	5.39	108.38	81.02	1.96	81.42
600		30.65	1.835	1.642	131.22	31.35	27.31	10.94	5.41	111.07	83.03	1.48	87.26
**LSD (P** ≤ **0.05)**													
**Treatment**		—	0.04	0.04	2.35	1.07	—	—	—	2.29	1.93	0.04	1.71
**CO**_**2**_**Level**		1.27	0.05	0.05	3.33	1.52	0.97	0.65	1.08	3.24	2.74	0.06	2.42
**CO**_**2**_**Level:Treatment**		—	0.08	0.08	4.71	2.15	—	—	—	4.58	3.87	0.09	3.42

Each value is mean of 5 replicates. Ambient ~375 ppm CO_2_.

Each value is mean of 5 replicates. Ambient ~375 ppm CO_2_.

**Table 3:** Effect of elevated levels of CO_2_ on colonization, conidia and fibrosin bodies of *S. fuliginea*, cuticular traits, physiological and biochemical parameters of cucumber inoculated with the powdery mildew fungus.

CO_2_ Concentrations (ppm)	*S. fuliginea*.	Colonization (%)	Trans piration (mmol H_2_O m-2 s^−1^)	Stomatal Conductance (mol m-2 s^−1^)	Phenolic contents (µg g^−1^ fresh leaf)	Carotenoids (µg g^−1^ fresh leaf)	Conidial size (µm)		Fibrosin bodies/conidia	Trichomes/cm^2^	Stomata/cm^2^	Size of stomatal aperture (µm)	Trichome length (µm)
							length	width					
Control (ambient)	Un inoculated		2.313	2.111	112.56	36.31				107.33	70.55	2.20	75.62
400			2.227	2.033	108.78	36.78				111.09	73.02	2.09	79.92
500			2.096	1.913	106.35	39.14				115.38	75.84	1.92	86.78
600			2.019	1.843	105.95	39.90				118.6	77.96	1.66	91.02
Control	Inoculated	48.55	2.505	2.286	129.12	33.37	25.32	10.63	5.03	100.25	65.89	2.46	69.14
400		50.25	2.419	2.208	134.23	33.44	26.13	10.97	5.19	103.79	68.22	2.38	72.48
500		55.83	2.285	2.086	135.70	35.84	26.21	11.00	5.21	108.4	71.26	2.07	79.26
600		53.89	2.211	2.018	136.54	36.60	26.28	11.03	5.22	111.09	73.02	1.98	82.24
**LSD (P ≤ 0.05)**													
**Treatment**		—	0.04	0.04	2.42	1.25	—	—	—	2.29	1.7	0.04	1.62
**CO**_**2**_**Level**		1.98	0.07	0.06	3.42	1.77	0.67	0.59	0.60	3.24	2.41	0.06	2.3
**CO**_**2**_**Level:Treatment**		—	0.09	0.09	4.84	2.51	—	—	—	4.59	3.41	0.08	3.24

Each value is mean of 5 replicates. Ambient ~375 ppm CO_2_.

**Table 4:** Effect of elevated levels of CO_2_ on colonization, conidia and fibrosin bodies of *S. fuliginea*, cuticular traits, physiological and biochemical parameters of pumpkin inoculated with the powdery mildew fungus.

CO_2_ Concentrations (ppm)	*S. fuliginea*.	Colonization (%)	Trans piration (mmol H_2_O m-2 s^−1^)	Stomatal Conductance (mol m-2 s^−1^)	Phenolic contents (µg g^−1^ fresh leaf)	Carotenoids (µg g^−1^ fresh leaf)	Conidial size (µm)		Fibrosin bodies/conidia	Trichomes /cm^2^	Stomata/cm^2^	Size of stomatal aperture (µm)	Trichome length (µm)
							length	width					
Control (ambient)	Un Inoculated		1.919	1.323	121.10	32.51				121.01	101.33	6.24	101.46
400			1.844	1.271	119.33	32.87				123.67	103.26	5.89	109.68
500			1.723	1.188	118.00	35.08				130.09	108.93	5.02	118.54
600			1.668	1.150	116.22	29.84				134.2	112.37	4.93	121.48
Control	Inoculated	53.27	2.084	1.437	128.55	35.83	27.34	11.52	5.91	112.66	94.34	6.98	97.36
400		55.13	2.009	1.385	136.36	29.97	28.21	11.89	6.10	117.02	97.99	6.27	99.45
500		61.05	1.888	1.302	138.20	32.09	28.30	11.92	6.12	122.22	102.34	5.84	106.23
600		59.29	1.833	1.263	140.10	32.77	28.38	11.96	6.13	125.25	104.88	5.32	111.54
**LSD (P ≤ 0.05)**													
**Treatment**		—	0.04	0.03	2.47	1.12	—	—	—	2.61	2.47	0.12	2.28
**CO**_**2**_**Level**		2.61	0.05	0.04	3.5	1.59	1.01	0.69	1.14	3.69	3.5	0.17	3.23
**CO**_**2**_**Level:Treatment**		—	0.08	0.06	4.95	2.24	—	—	—	5.22	4.95	0.24	4.57

Each value is mean of 5 replicates. Ambient ~375 ppm CO_2_.

**Table 5:** Effect of elevated levels of CO_2_ on colonization, conidia and fibrosin bodies of *S. fuliginea*, cuticular traits, physiological and biochemical parameters of sponge gourd inoculated with the powdery mildew fungus.

CO_2_ Concentrations (ppm)	*S. fuliginea*.	Colonization (%)	Trans piration (mmol H_2_O m^−2^ s^−1^)	Stomatal Conductance (mol m^−2^ s^−1^)	Phenolic contents (µg g^−1^ fresh leaf)	Carotenoids (µg g^−1^ fresh leaf)	Conidial size (µm)		Fibrosin bodies/conidia	Trichomes /cm^2^	Stomata/cm^2^	Size of stomatal aperture (µm)	Trichome length (µm)
							length	width					
Control (ambient)	Un inoculated		3.231	2.213	116.31	34.53				126.37	107.43	4.82	82.8
400			3.376	2.313	115.65	34.94				128.77	109.47	4.01	88.54
500			3.625	2.483	113.70	37.36				135.97	115.59	3.87	96.36
600			3.719	2.547	116.28	38.16				140.02	119.03	3.08	102.45
Control	Inoculated	49.63	3.525	2.414	132.55	31.32	29.03	11.08	6.02	118.16	100.45	5.41	78.47
400		51.37	3.376	2.313	138.11	31.80	29.96	11.43	6.21	121.82	103.56	5.01	80.5
500		56.18	3.131	2.144	141.40	34.08	30.05	11.47	6.23	128.14	108.93	4.88	87.48
600		55.09	3.040	2.082	143.23	34.81	30.13	11.50	6.25	131.17	111.51	4.21	94.57
**LSD (P** ≤ **0.05)**													
**Treatment**		—	0.10	0.07	3.58	1.69	—	—	—	3.82	3.67	0.13	2.61
**CO**_**2**_**Level**		2.31	0.07	0.05	2.53	1.19	1.07	0.74	0.71	2.70	2.59	0.92	1.85
**CO**_**2**_**Level:Treatment**		—	0.14	0.10	5.07	2.39	—	—	—	5.4	5.19	0.18	3.7

Each value is mean of 5 replicates. Ambient ~375 ppm CO_2_.

### Foliar colonization by *S. fuliginea*, conidia size, and fibrosin bodies

The colonization by *S. fuliginea* was not observed on any cucurbit species which were not inoculated with the fungus. Among the cucurbit species inoculated with *S. fuliginea*, highest mildew colonization was recorded on pumpkin (53%) and lowest on bitter gourd (14%, Tables 1–5). The order of overall colonization by *S. fuliginea* on cucurbit species was pumpkin > sponge gourd > cucumber > bottle gourd > bitter gourd (Tables 1–5). With regard to CO_2_ concentrations, significantly greater mildew colonization was recorded at 500 ppm followed by 600 and 400 ppm CO_2_ over control (Tables 1–5). According to ANOVA, the individual and combined effects of CO_2_ and cucurbits were significant for the colonization by *S. fuliginea* at P ≤ 0.001.

Size of conidia of *S. fuliginea* increased with increasing CO_2_ concentration. Maximum enhancement in the size of conidia (length and width) was observed at 600 ppm concentration of CO_2_, followed by 500, 400 and 375 ppm (ambient) concentration. However, the number of fibrosin bodies per conidia were not affected by CO_2_ exposures. Similarly, an effect of CO_2_ exposures on conidia germination was not recorded, and forked germ tube was uniformly developed when conidia from the exposed plants were incubated.

### Number of stomata and trichomes, size of stomatal aperture and length of trichomes

The number of stomata and trichomes per unit leaf surface increased with increasing CO_2_ concentration and a maximum count was recorded at 600 ppm concentration (P ≤ 0.05; Tables 1–5). The plants inoculated with powdery mildew fungus showed a decrease in the number of stomata and trichomes. However, in the inoculated plants stomata counts increased with an increase in the gas concentration (P ≤ 0.05). In the CO_2_ treatments, the overall density of stomata and trichomes on the leaf surface in inoculated plants was significantly less than uninoculated plants at P ≤ 0.05 (Tables 1–5)

Size of the stomatal aperture was found to be negatively correlated with CO_2_ concentration, and stomata tended to remain partially closed at higher CO_2_ concentration (Tables 1–5). Therefore, size of stomatal aperture was decreased with increasing concentration of the gas, and the smallest stomatal aperture was recorded at 600 ppm CO_2_. On the other hand, in inoculated plants, the size of stomatal aperture was increased but decreased upon exposure to CO_2_. The length of trichomes increased with increasing CO_2_ concentration and longer trichomes were recorded at 600 ppm CO_2_ (P ≤ 0.05; Tables 1–5). Whereas, the plants inoculated with the powdery mildew fungus showed a reduction in the trichome length (P ≤ 0.05). However, in the plants exposed to CO_2_ and inoculated with *S. fuliginea*, the stomatal density was maximum at 600 ppm concentration followed by 500, 400 and 375 ppm concentrations (Tables 1–5).

### Foliar salicylic acid, phenolic compounds, and pigments

**Figure 6:**
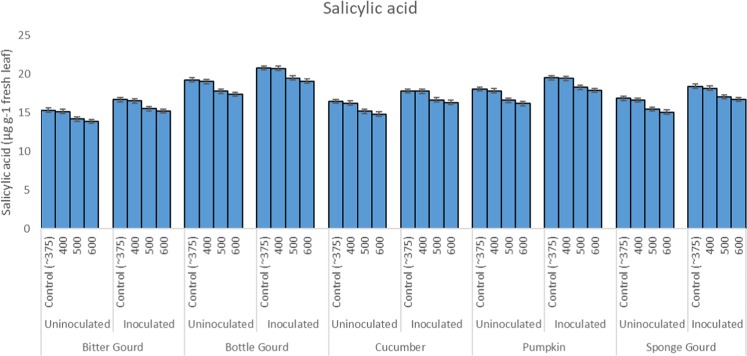
Bar diagram showing the effect of CO_2_ exposures and *S. fuliginea* inoculation singly and concomitantly on the salicylic acid content of five cucurbits.

Intermittent exposures of plants to 600 ppm CO_2_ resulted in a significant decrease in the foliar salicylic acid content (SAC) and total phenolic contents (TPC) of the cucurbit species in comparison to the control or 400 ppm CO_2_ (P ≤ 0.05; Tables 1–5; Fig. 6). Inoculation with *S. fuliginea* caused a significant increase (P ≤ 0.05) in the SAC and TPC of all five cucurbits as compared to control (P ≤ 0.05). The combined effects of CO_2_ and *S. fuliginea* on SAC and TPC was near to the sum of the increase or decrease caused by *S. fuliginea* and CO_2_ individually. However, at 600 ppm CO_2_ concentration, a further decrease in the SAC in all five cucurbits was recorded. The single and combined effects of CO_2_ and *S. fuliginea* on the SAC were found significant at P ≤ 0.001 (Fig. 6).

**Figure 7:**
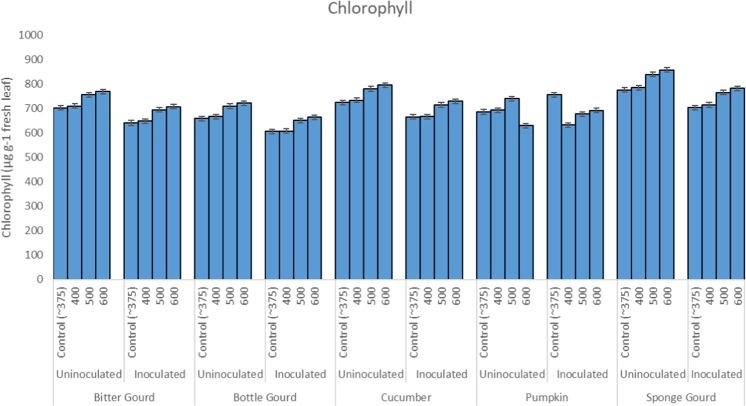
Bar diagram showing effect of CO_2_ exposures and *S. fuliginea* inoculation singly and concomitantly on the chlorophyll content of five cucurbits.

The Chlorophyll a content of leaves was found to be increased with the increase of CO_2_ concentration, and maximum enhancement was recorded at 600 ppm CO_2_ (Fig. 7). The leaf carotenoids were also increased due to exposure to 500 and 600 ppm CO_2_ as compared to the respective controls (Fig. 7). Inoculation with *S. fuliginea* resulted in a substantial suppression in the chlorophyll and carotenoid contents in all five cucurbits over control (Fig. 7). Species-wise, the order of reduction in chlorophylls and carotenoids was: pumpkin > bottle gourd > sponge gourd > cucumber > bitter gourd. Leaf pigments of cucurbits inoculated with *S. fuliginea* and exposed to 500 or 600 ppm CO_2_ also showed significant increment in comparison to fungus inoculated respective controls (Tables 1–5; Fig. 7). According to ANOVA single and interaction effects of CO_2_ and *S. fuliginea* were found significant for each pigment at P ≤ 0.001.

### Stomatal conductance, photosynthesis, and transpiration rate

**Figure 8:**
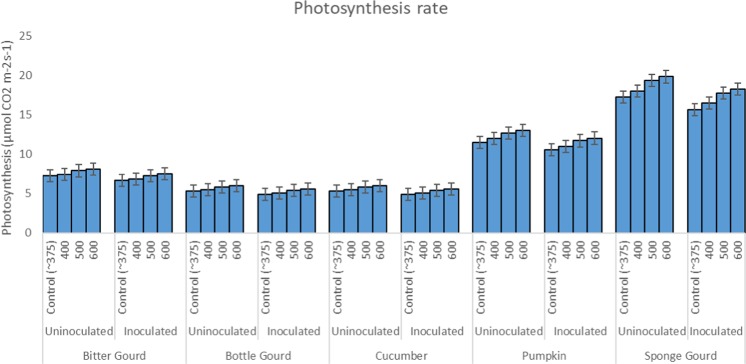
Bar diagram showing effect of CO_2_ exposures and *S. fuliginea* inoculation singly and concomitantly on the rate of photosynthesis of five cucurbits.

Rate of photosynthesis in inoculated cucurbit species was increased with the increase of CO_2_ concentrations (Fig. 8). Highest photosynthesis rate, 11–14% greater than the control, was recorded at 600 ppm CO_2_ (12–13%). The photosynthesis rate at 500 ppm CO_2_ was 8–10% ie., 3–4% lesser than 600 ppm but significantly greater than 400 ppm CO_2_ (Fig. 8). The difference between 400 ppm CO_2_ and ambient control (~375 ppm) was not significant at P ≤ 0.05. On the other hand, the rate of transpiration and stomatal conductance decreased significantly with increasing CO_2_ levels as compared to the ambient control (Tables 1–5). Statistically, the trend for decrease in transpiration or stomatal conductance was more or less similar to the increase in the photosynthesis rate at P ≤ 0.05. Plants inoculated with *S. fuliginea* showed significant decrease in the photosynthesis rate, and subsequent increase in transpiration and stomatal conductance as compared to the uninoculated plants. The decrease in the photosynthesis rate and increase in transpiration in different cucurbits due to powdery mildew were in the order of pumpkin > sponge gourd > cucumber > bottle gourd > bitter gourd (Tables 1–5; Fig. 8). The response pattern of stomatal conductance was statistically identical to the transpiration rate.

The response of the physiological parameters of the cucurbit species to concomitant inoculation and exposure was more or less similar to the effects observed for the individual effects of *S. fuliginea* and CO_2_ concentrations. The rate of photosynthesis in the fungus plus CO_2_ treatments increased with increasing level of CO_2_ (400–600 ppm) in comparison to fungus inoculated control, but it was significantly less than the CO_2_ exposed plants without fungus inoculations (P ≤ 0.05). Likewise, the rate of transpiration and stomatal conductance decreased with the increase of CO_2_ level.

**Figure 9:**
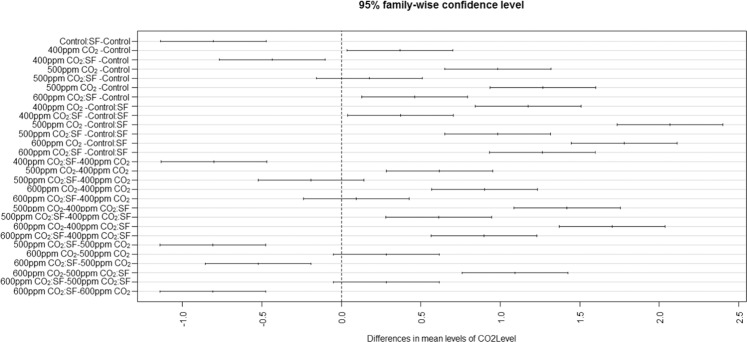
Effect of intermittent exposures of CO_2_ at 375 (ambient), 400, 500 and 600 ppm and *S. fuliginea* on the photosynthesis rate in cucurbits. The contrasts situated on the left side of the line of no difference show negative interaction (antagonistic), whereas the contrasts located on the right side of the line of no difference represent positive interaction (synergistic). The contrast on the farthest location shows highest interaction effects.

The Fig. 9 prepared on orthogonal contrasts between CO_2_ levels and fungus on the photosynthesis rates shows mostly significant interaction between CO_2_ and *S. fuliginea* as only a few contrasts lie on the broken vertical line of no difference. Maximum synergistic interaction with regard to suppression in the photosynthesis rate was observed for 600 ppm CO_2_: *S. fuliginea*, as this contrast was situated at the farthest location on the right side of the line of no difference (Fig. 9). Individual and combined effects of CO_2_ and *S. fuliginea* were found significant at P ≤ 0.001.

## Discussion

The cucurbit plants inoculated with conidia of *S. fuliginea* were found susceptible to the fungus and developed characteristic symptoms of powdery mildew. However, bitter gourd expressed moderate susceptibility to the fungus. The symptom of powdery mildew appeared first on the older leaves as circular, small and white powdery mass (conidia and mycelia). Cucurbit plants are one of the most susceptible hosts to *S. fuliginea*^26^ and formation of powdery mildew colonies on leaves, in particular, is a characteristic symptom of infection by this fungus^27^. The number and size of the mildew colonies later increased and eventually coalesced covering most of the leaf surface. The severity of disease, however, varied greatly among the five cucurbit species. Khan^28^ have reported that although most of the cucurbits are hosts to *S. fuliginea* or *Erysiphe*, their relative susceptibility varies greatly for both the genera and bitter gourd expresses lesser susceptibility to the fungus, as observed in the present paper.

The inoculation with powdery mildew fungus resulted in a significant suppression in the photosynthesis rate, leaf pigments, plant growth and increase in the salicylic acid and phenol contents, transpiration rate and stomatal conductance over control. Development of the mildew colonies on the leaf surface would have reduced the incidence of light to the mesophyll cells evidenced by a significant reduction in the leaf chlorophyll and carotenoids^29^. Reduction in the photosynthetic pigments is directly correlated with photosynthetic efficiency of the leaf surface^29^. As a result, the photosynthesis rate decreased significantly in all five infected cucurbit species. Further, the photosynthesis rate is inversely proportional to the transpiration rate and stomatal conductance^30^. Moreover, the fungus colonization would have caused partial plugging of stomata cavity or pore by hyphae and/or spores of the fungus. As a result, transpiration rates were invariably decreased in the fungus infected plants. Formation of mildew cover over the leaf surface would have apparently hampered the incidence of light to stomata. Salicylic acid and phenolic are the biochemicals that trigger host defence^31^, and their synthesis is accelerated in response to any biotic or abiotic stress. Pathogenic infection is one of the important biotic agents that may activate the synthesis of salicylic acid^31^ and phenolic compounds^32^, as recorded in the present study.

Exposure of cucurbit species to 500 and 600 ppm CO_2_ caused significant enhancement in the photosynthesis rate, leaf pigments and plant growth, whereas a decrease in salicylic acid and phenolic contents of plants. Plants utilize CO_2_ gas in the process of photosynthesis. The elevated levels of CO_2_ would have created and maintained a higher concentration of CO_2_ in the chloroplast leading to a decrease in the photorespiration. Photorespiration is a process of wasteful release of CO_2_ that takes place due to the increase of O_2_ concentration in the chloroplast that causes conversion of carboxylate enzyme into oxygenase in C_3_ plants. Elevated levels of CO_2_ such as 500–600 ppm have been reported to substantially check photorespiration in C_3_ plants by reducing O_2_ concentration and increasing CO_2_ concentration in the mesophyll cells. Since cucurbits are typical C_3_ plants, 500–600 ppm induced 8–14% increase in photosynthesis in the five cucurbit species tested. The 800 ± 20 ppm CO_2_ exposures are reported to increase photosynthesis rate by 16.9%^33^ and ~700 ppm by 15–25%^34^. In contrast to photosynthesis, the elevated levels of CO_2_ decreased the rate of transpiration, stomatal conductance, salicylic acid and phenol contents significantly (P ≤ 0.05). These physiological and biochemical responses observed in the present study are in accordance with previous researches^30^.

Intermittent CO_2_ exposures predisposed the cucurbit plants to the infection by *S. fuliginea* as well as influenced host-pathogen relationship. Overall, elevated levels of CO_2_ (500–600 ppm) significantly promoted the infection of *S. fuliginea* in all five cucurbit species. Further, bitter gourd developed disease severity of 16%, but under exposure to 500–600 ppm, the mildew severity rose to more than double *i.e*., 35–39%, demonstrating elevated CO_2_ level predisposed the plants, and as a result, moderate resistance reaction of bitter gourd to *S. fuliginea* was modified into a susceptible reaction. The fungus is an ectoparasite and its spores colonize leaf surface^35,36^. It is quite possible that greater foliage production in terms of leaf area and leaf thickening induced by 500–600 ppm CO_2_ would have apparently provided a better substrate in terms of area, succulence and/or nutrition that significantly promoted the colonization of *S. fuliginea* and disease severity^35,37^. Lake and Wade^15^ have reported that plants became prone to powdery mildew disease when exposed to CO_2_ at 800 ppm. The present study has revealed that under 500–600 ppm CO_2_ exposures, plant growth of cucurbits may be improved by 9–17%, but at the same time plants may become more susceptible to *S. fuliginea*, and the disease severity may increase significantly as recorded for all five cucurbit species in the present study.

## Conclusion

The study has demonstrated that elevated CO_2_ levels on cucurbits may influence the host-parasite relationship of powdery mildew fungus. The intermittent exposures of plants to 500 and 600 ppm CO_2_ enhanced the plant growth and biomass production of five cucurbit species tested by 9–14% and 10–17% respectively. The CO_2_ exposures also enhanced the susceptibility of the cucurbits to *S. fuliginea*, leading to 67% and 59% increase in the disease severity at 500 and 600 ppm, respectively. The bitter gourd plants demonstrated moderate resistance to mildew fungus with 16% disease severity, but the severity increased to 35–39% upon exposure to 500–600 ppm CO_2_. Synergistic interaction between powdery mildew fungus and 400–500 ppm CO_2_ was recorded which is highly alarming as 400+ ppm concentration is relevant to existing ambient CO_2_ levels^38^. A concentration of 400–500 ppm CO_2_ may occur in the environments close to petroleum refineries, coal-fired thermal power plants, busy highways or other CO_2_ sources^17^.
